# Solid Lipid Nanoparticles as Formulative Strategy to Increase Oral Permeation of a Molecule Active in Multidrug-Resistant Tuberculosis Management

**DOI:** 10.3390/pharmaceutics12121132

**Published:** 2020-11-24

**Authors:** Antonella Obinu, Elena Piera Porcu, Sandra Piras, Roberta Ibba, Antonio Carta, Paola Molicotti, Rossana Migheli, Alessandro Dalpiaz, Luca Ferraro, Giovanna Rassu, Elisabetta Gavini, Paolo Giunchedi

**Affiliations:** 1 Department of Chemistry and Pharmacy, University of Sassari, via Muroni 23/a, 07100 Sassari, Italy; aobinu@uniss.it (A.O.); elena.piera1988@gmail.com (E.P.P.); piras@uniss.it (S.P.); ribba@uniss.it (R.I.); acarta@uniss.it (A.C.); grassu@uniss.it (G.R.); pgiunc@uniss.it (P.G.); 2 Department of Biomedical Sciences, University of Sassari, Viale San Pietro, 07100 Sassari, Italy; molicott@uniss.it; 3 Department of Clinical and Experimental Medicine, University of Sassari, viale San Pietro 43/b, 07100 Sassari, Italy; rmigheli@uniss.it; 4 Department of Chemical and Pharmaceutical Sciences, University of Ferrara, via Fossato di Mortara 19, 44121 Ferrara, Italy; dla@unife.it; 5 Department of Life Sciences and Biotechnology, University of Ferrara, via Borsari 46, 44121 Ferrara, Italy; frl@unife.it

**Keywords:** solid lipid nanoparticles, drug-resistant tuberculosis, oral permeation, antitubercular activity, Witepsol^@^, Gelucire^@^

## Abstract

The role of mycobacterial efflux pumps in drug-resistant tuberculosis has been widely reported. Recently, a new compound, named SS13, has been synthesized, and its activity as a potential efflux inhibitor has been demonstrated. In this work, the chemical–physical properties of the SS13 were investigated; furthermore, a formulative study aimed to develop a formulation suitable for oral administration was performed. SS13 shows nonintrinsic antitubercular activity, but it increases the antitubercular activity of all the tested drugs on several strains. SS13 is insoluble in different simulated gastrointestinal media; thus, its oral absorption could be limited. Solid lipid nanoparticles (SLNs) were, therefore, developed by using two different lipids, Witepsol and/or Gelucire. Nanoparticles, having a particle size (range of 200–450 nm with regards to the formulation composition) suitable for intestinal absorption, are able to load SS13 and to improve its permeation through the intestinal mucosa compared to the pure compound. The cytotoxicity is influenced by the concentration of nanoparticles administered. These promising results support the potential application of these nanocarriers for increasing the oral permeation of SS13 in multidrug-resistant tuberculosis management.

## 1. Introduction

Tuberculosis (TB) is a lung infection caused by *Mycobacterium tuberculosis*. The World Health Organization reported that, after HIV, TB is the second leading cause of death induced by one infectious agent. Every year, active TB affects about 9 million people and causes almost 2 million deaths [1]. Particularly, as reported in the Global Tuberculosis Report 2019 [2], in 2018, 10.0 million people developed TB, and 1.5 million people died from this infection. Regularly, the World Health Organization approves protocols for TB management [3]; however, even though the most used therapies for drug-sensitive TB are highly efficient, more effective treatments are necessary to decrease the infection diffusion. The new cases of multidrug-resistant TB (MDR-TB) (resistance to isoniazid and rifampicin) and of extensively drug-resistant TB (XDR-TB) (resistance to isoniazid and rifampicin plus fluoroquinolones like ofloxacin or moxifloxacin, along with resistance to one of the injectable second-line antibiotics such as amikacin, capreomycin or kanamycin) continue to spread worldwide [4]. In 2018, 484,000 people developed resistance to rifampicin, and 80% of those showed MDR-TB [5]. Most of the drug-resistant *M. tuberculosis* strains show a spontaneous gene mutation that provides resistance to the most employed anti-TB agents. Mutations in the gene coding for the drug target (e.g., RNA polymerase, topoisomerases or ribosomal proteins) cause a reduction of drug affinity for the target [6]. The repeated exposure of *M. tuberculosis* cells to drugs results in an upregulation of the efflux pump system genes, leading to an increased number of efflux pumps on the cell membrane [7]. Treatment outcomes for MDR and XDR-TB are still poor, resulting in high rates of deaths. This highlights the urgent need to improve the development of innovative treatment regimens by introducing new drugs that may overcome the growing diffusion of MDR/XDR-TB, such as new compounds that elude the target mutation [8] or efflux-pump inhibitors (EPIs) that revert the drug resistance. Efflux pump overexpression reduces the clinical efficacy of antibiotics by decreasing their intracellular concentration and is thus involved in the development of drug resistance by *M. tuberculosis* [9]. Consequently, efflux pumps represent an interesting target in antituberculosis research.

Recently, a new class of compounds has been synthesized and investigated as a potential efflux pump inhibitor; this class is based on the phenoxymethylquinoxalines, which have already proven capable of restoring or potentiating the anticancer activity of antitumoral drugs in tumor cell lines [10,11]. On the basis of the results obtained, novel derivatives (EPIs) were designed to be more selective against *M. tuberculosis* efflux pumps to restore the activity of anti-TB drugs by reducing their cell extrusion. The synthetic route and the chemical structure of EPIs cannot be reported here as they are in the process of patent protection. After preliminary microbiological studies, a promising compound labeled SS13 was identified, and the effect of this molecule on several strains (both commercial reference and clinical strains) of *M. tuberculosis* when associated with antibiotics employed in the therapy of TB was evaluated. Moreover, SS13 was characterized by the point of view of chemical–physical properties and its suitability for oral administration. The challenge of developing an appropriate delivery system to increase SS13 oral bioavailability was addressed by exploiting by nanotechnology strategy; the small size of these systems increases the specific surface area, allowing fast drug dissolution and increased contact area with the epithelial surface [12,13]. Furthermore, because the nanoparticles should be picked up by intestinal cells, the toxicity was evaluated on human colorectal adenocarcinoma (Caco-2) cells as an epithelial intestinal model [14].

Among the various types of nanoparticles studied, the solid lipid nanoparticles (SLNs) seem to be encouraging systems for oral delivery [15] due to many advantages like nontoxicity, stability and high biocompatibility [16]. Sufficient literature data displayed that the oral bioavailability of some substances, for example, curcumin [17], paclitaxel [18] and sorafenib [19], can be increased when encapsulated in SLNs. So far, the use of Witepsol as a constituent of SLNs has been described in several literature works [20,21,22]. The pharmaceutical industry used Witepsol as an excipient, but it has also been investigated for oral application to mask the unpleasant tastes and to improve drug absorption in the oral formulation [23,24]. Witepsol-based SLNs were here prepared; also, SLNs consisting of a lipid mixture of Witepsol and Gelucire were studied. Gelucire is a lipid excipient derived from a mixture of glyceride-based materials and esters of polyethylene glycol widely studied to enhance solubility, stability and dissolution rate after oral administration [25]. The improvement of bioavailability associated with this excipient can be due to its excellent surface-active property that increases drug solubilization, thus favoring absorption [26].

## 2. Materials and Methods

### 2.1. Materials

Witepsol E85 (W) was supplied by Cremer Oleo (Hamburg, Germany); Gelucire 44/14 (G) was kindly provided by Gattefossé SAS (Saint-Priest, France). Ethanol, polyvinylalcohol (PVA), dichloromethane (DCM) and octan-1-ol were acquired from Sigma-Aldrich (St. Louis, MO, USA). Acetonitrile and dimethylsulfoxide (DMSO) were acquired from Merck (Darmstadt, Germany). Ultrapure bidistilled water was obtained by a MilliQ R4 system, Millipore (Milan, Italy). The fresh intestine was taken from a local slaughterhouse (Forma s.r.l; CE IT D2L5B (Regulation EC 853/2004)). Fetal bovine serum (FBS), Dulbecco’s modified Eagle’s medium (DMEM/F12, HEPES, no phenol red) and streptomycin–penicillin were acquired from Life Technologies Italia (Monza, Italy). Phosphate-buffered saline (PBS, NaCl 0.138 M; KCl 0.0027 M; pH 7.4, at 25 °C), Tween80 and 3-(4,5-dimethyl-thiazol-2-yl)-2,5,diphenyltetrazoliumbromide (MTT, 97.5%) were obtained from Sigma-Aldrich (Milan, Italy).

### 2.2. Antimycobacterial Activity Assay

The antimycobacterial activity was determined by resazurin microtiter assay (REMA) assay against *M. tuberculosis* H37Rv strain (ATCC 27294) and the following clinical isolates from Italian hospitals: 952, 1097, 1670, 512, 600, 368, 1762, 571 and 1120. REMA assays were performed in sterile 96-well microtiter plates with round-bottom wells, sealed in a plastic bag. Mycobacteria were cultured in Middlebrook 7H11 medium supplemented with OADC. The bacterial suspensions (at the standard turbidity of 1 McFarland) were diluted 1:10, 1:100 and 1:10,000 with Middlebrook 7H9 and inoculated in duplicate Middlebrook 7H11 plates containing serially diluted concentrations of the drugs (streptomycin, isoniazid, rifampicin, ethambutol and ciprofloxacin) and test compound (SS13). Final bacterial inocula were approximately 1–5 × 10^5^ CFU/mL. Plates were incubated at 37 °C and were read after 72 h. Once the final visual reading was performed, 30 μL of 0.02% resazurin was added to each well before reincubation overnight. Bacterial growth was measured by the change in color from blue (oxidized state) to pink (reduced), and minimal inhibitory concentration (MIC) was defined as the lowest drug concentration that prevented the color change and, therefore, the mycobacteria growth.

### 2.3. Determination of SS13 Solubility and Partition Coefficient

An opportune amount of SS13 (2 mg) was dispersed in 5 mL of different media (buffer pH 1.2, 6.8 and 7.4 and bidistilled water). The suspensions were placed into an incubator shaker apparatus, SKI 4 Shaker Incubator (Argo Lab, Carpi, Italy), for 72 h at 150 rpm and 37 °C. The solubility was measured by the saturation shake-flask method [27]. After this time, 1 mL of each suspension was centrifuged twice with a Hettich Mikro 120 centrifuge (Tuttlingen, Germany) for 10 min at 14,000 rpm. The analysis of obtained supernatants was carried out with a UV spectrophotometer (Shimadzu UV-1800, Kyoto, Japan) (λ = 260 nm). The solubility values were determined by using previously prepared calibration curves: bidistilled water, y = 86.371x + 0.0499; buffer pH 1.2, y = 84.229x + 0.0362; buffer pH 6.8, y = 65.400x + 0.1239; buffer pH 7.4, y = 67.371x + 0.0775.

For the determination of partition coefficient (Log P), shake-flask method was used [28]; 5 mg of SS13 was dispersed in 5 mL of bidistilled water, and 5 mL of octan-1-ol was added to the suspension. Dispersions tubes were rotated at 5 rpm for 72 h using a tube rotator in order to promote contact between the two phases. Drug concentration in each phase was evaluated by UV spectroscopy (λ = 260 nm) using the following calibration curve: y = 149,098.15x − 5399.13.

### 2.4. Preparation of SLNs

Two SLN formulations (SLN-W and SLN-G), characterized by a different lipid composition (W for SLN-W and a mixture of W and G for SLN-G), were obtained employing a modified solvent emulsification–evaporation method [22]. Briefly, for SLN-W preparation, W (200 mg) was dissolved in 2 mL of DCM, and 6.4 mg of SS13 was dispersed in this organic solution. Then, 10 mL of PVA aqueous solution (2% *w*/*v*) was poured into the organic phase under magnetic stirring. The resulting pre-emulsion was homogenized with a probe sonicator Vibra Cell, VC 50 (Sonics and Materials, Danbury, CT, USA) for 30 s at 70% amplitude. To remove the organic solvent, the obtained emulsion was stirred for 3 h at room temperature. The formulation was centrifuge at 4000 rpm for 5 min in order to precipitate SS13 eventually free. SLN-G was prepared as reported above, but the organic phase consisted of a lipid mixture of W and G (mass ratio 5:4) and SS13 (6.4 mg) in 4 mL of DCM. The pre-emulsion was sonicated for 90 s at 70% amplitude. This sonication time was chosen on the basis of formulative studies in which different times were tested (30, 60 and 90 s). In these studies, to select the best sonication time, the particle size and the polydispersity index (PDI) of obtained SLNs were determined as reported in Section 2.6.

The same procedures were followed to prepare unloaded SLNs (SLN-Wb and SLN-Gb).

During this phase, the preparation of SLNs containing only G as lipid excipient was also evaluated.

### 2.5. Evaluation of SS13 Loading

In order to quantify the loading of SS13, 50 µL of formulation (SLN-W and SLN-G) was added to acetonitrile (950 µL) for extracting the molecule loaded. Only one extraction was sufficient. The obtained suspension was vortexed for 1 min and centrifuged at 14,000 rpm for 10 min. After filtration on 0.2 μm regenerated cellulose membrane filters (Albet Labscience, Dassel, Germany), the samples were analyzed using the HPLC method described above. The SS13 loading (DL) (%) was calculated according to the following equation:

(1) $$\mathrm{DL}\left(\%\right)=\frac{\mathrm{amount}\;\mathrm{of}\;\mathrm{SS}13\;\mathrm{in}\;\mathrm{SLN}\;}{\mathrm{theretical}\;\mathrm{amount}\;\mathrm{of}\;\mathrm{SS}13\;\mathrm{used}}\times 100$$

The amount of SS13 was evaluated using a modified high-performance liquid chromatography (HPLC) method. A Varian HPLC–DAD system (Palo Alto, CA, USA) consisting of two ProStar 210 pumps, a ProStar 410 autosampler and a DAD Varian 330 detector was employed. The chromatographic separation was done on a Nucleosil C18 column (100 mm × 4.6 mm, Machery-Nagel, Düren, Germany). The binary mobile phase, a solution of acetonitrile and water at 90:10 *v*/*v*, was eluted at a flow rate of 1.5 mL/min at room temperature. Before use, the mobile phase was filtered by 0.22 µm cellulose regenerated membrane filters (Sartorius, Goettingen, Germany). The volume of injection was 10 μL and the elution time was 3 min. Peak heights rather than areas in the chromatography were recorded and measured at 254 nm. Concentrations of SS13 were calculated by interpolation with a previously prepared calibration curve (y = 488,502.8x − 147,055; R^2^ = 0.9998), obtained using SS13 standard solutions. The stock standard solution of SS13 was obtained by solubilizing the exactly weighed molecule in DMSO to obtain a concentration of 1 mg/mL. The standard solutions used to prepare the calibration curve were obtained by diluting the 1 mg/mL stock solution at 2.5–20.0 μg/mL with acetonitrile.

### 2.6. Analysis of Particle Size, Polydispersity and Zeta Potential of Loaded and Unloaded SLNs

Dimensional properties and PDI of unloaded and loaded SLNs were determined with a Coulter nanosizer N5 (Beckman Coulter Inc., Miami, FL, USA) by photon correlation spectroscopy. The equipment requires an exact concentration of the sample (range 5 × 10^4^ to 1 × 10^6^ counts/s); therefore, before every analysis, the SLN suspensions (20 µL) were diluted with bidistilled water. Triplicate SLN formulations were prepared and each sample was analyzed three times (*n* = 9).

Zeta potential of samples was evaluated by electrophoretic mobility using a 90 Plus instrument (Brookhaven, NY, USA). SLN dispersions were diluted with deionized distilled water and 0.1 mM KCl3. For the analysis, the formulations were positioned in the electrophoretic cell, where an electric field of about 15 V/cm was applied.

### 2.7. Morphological Analysis

The morphology and surface features of SLNs were examined by transmission electron microscopy (TEM,) and atomic force microscopy (AFM,).

#### 2.7.1. Transmission Electron Microscopy

The morphology of SLN-W, SLN-G and corresponding unloaded nanoparticles was examined by a Tecnai G2 F20 Twin TMP (FEI Company, Dawson Creek, Hillsboro, OR, USA). For the analysis, an exact amount of SLN formulation (20 µL) was deposited on carbon film copper grids (200 mesh). To achieve proper drying, the grids were left overnight at room temperature. Then, 10 µL of uranyl acetate solution (1 M) was dropped on the grids which were dried again overnight. The negatively stained SLNs were observed by TEM, and data and images were analyzed with a Digital Micrograph from Gatan and with TIA software.

#### 2.7.2. Atomic Force Microscopy

An atomic force microscope MFP-3D (Asylum Research, Oxford Instruments Company, Santa Barbara, CA, USA) was used to determine the topography of the SLNs. The tips were Nanosensors model EFM (k1/4 2N m_1, Ptlr5 coating). For this, SLN formulations (40 µL) (SLN-W, SLN-G, SLN-Wb and SLN-Wb) were dropped on a microscope glass slide and stored at room temperature overnight. The Igor Pro6.3.4.1 MFP3D Template software was used to obtain the images.

### 2.8. Fourier Transform Infrared Spectrometry Analysis

Fourier transform infrared spectrometry (FTIR) was employed to determine the structural characterization of samples (SLN-Wb, SLN-Gb, SLN-W, SLN-G and SS13, as well as the physical mixtures of SLN-Wb with SS13 and SLN-Gb with SS13). To obtain a powder material, the formulations were stored at room temperature overnight, and then KBr disks were prepared. FTIR spectra were acquired with a Nicolet Avatar 320 FTIR spectrometer (Nicolet Instrument Corporation, Madison, WI, USA).

### 2.9. X-ray Analysis

X-ray diffraction (XRD) measurements were made with a SmartLab X-ray diffractometer (Rigaku Europe SE, Neu-Isenburg, Germany) equipped with a rotating copper anode working at a power of 40 kV and 100 mA.

### 2.10. Physical Stability Studies

The influence of time and storing conditions on the physical stability of loaded and SS13-loaded SLNs was evaluated. The formulations were stored at 4 and 25 °C, and particle size and PDI were evaluated at 1, 7, 15 and 30 days. The dimensional properties of SLNs were compared with those determined after the nanoparticle preparation. In addition, the resistance of the SLNs to the gastric condition was evaluated. For this purpose, 1 mL of formulations (SLN-W and SLN-G) was dispersed into 9 mL of hydrochloric acid buffer pH 1.2. The dispersions were placed into the SKI 4 Shaker Incubator (Argo Lab, Carpi, Italy) for 2 h at 70 rpm. After this time, the particle size of the SLNs was determined.

### 2.11. In Vitro SS13 Release Study

SS13 solubility in PBS containing 0.2% *v*/*v* Tween80 (Tween/PBS) or PBS containing 50% *v*/*v* FBS (FBS/PBS) was determined [29]: an excess of SS13 (2.0 mg) in 5 mL of each medium was incubated at 37 °C for 72 h at constant agitation in the SKI 4 Shaker Incubator (Argo Lab, Carpi, Italy). Samples (1 mL) were centrifuged twice at 14,000 rpm for 10 min to separate a supernatant. Dissolved SS13 in the supernatant was analyzed with HPLC as described in Section 2.5. Before HPLC analysis, 300 μL of acetonitrile was added to FBS/PBS supernatant, which was then centrifuged at 14,000 rpm for 5 min. Solubility studies were carried out in triplicate (*n* = 3).

In vitro release studies were performed using the shaker incubator at 37 ± 0.5 °C [30]. To detect the amount of SS13 released from formulations, an amount of SLN-W or SLN-G (equivalent to 0.12 mg of SS13) was placed in 15 mL of FBS/PBS, creating a condition close to the solubility, and stirred at 80 rpm for 24 h. At predetermined time points, the medium (0.5 mL) was withdrawn by a syringe equipped with a filter (Corning syringe filters, regenerated cellulose membrane, diameter 4 mm, pore size 0.2 μm; Merck KGaA, Darmstadt, Germany). The release medium was replaced with the corresponding volume of fresh FBS/PBS, and the syringe filter was washed. Three hundred microliters of acetonitrile was added to samples, which were centrifuged at 14,000 rpm for 5 min and then injected into HPLC. The same test was carried out in FBS/PBS using an SS13 dispersion in 2% w/v aqueous PVA as control. In vitro release studies were performed in triplicate (*n* = 3).

### 2.12. Ex Vivo Permeation Studies on Intestinal Mucosa

**Figure 1:**
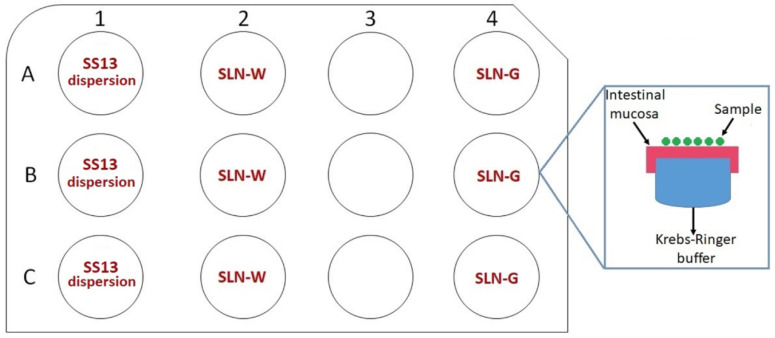
Schematic representation of plates used for ex vivo permeation test. The test was carried out using three different plates, one for each time point. Blue box reports the arrangement of intestinal mucosa and samples over each well. All samples were tested in triplicate (*n* = 3).

Ex vivo permeation of SLN-W and SLN-G was evaluated on excised pig intestinal mucosa using an SS13 dispersion in 2% *w*/*v* aqueous PVA as control. Before the test, intestinal fragments were cut along the mesenteric line and the tunica serosa was removed [31,32]. The experiment was carried out using three (one for each time point: 30, 60 and 120 min) 12-well cell culture plates suitably modified (project INCREASE SARDINIA 2016-17, protocol number 31351, University of Sassari). On each plate, all samples were tested in triplicate. The wells were filled with a Krebs-Ringer bicarbonate buffer (pH 7.4), and the intestinal mucosa was put on the plates; particular care was taken to prevent the air bubble formation at the mucosa–buffer interface. An exact amount of fresh samples (100 μL, corresponding to 0.06 mg of SS13) was positioned on the mucosal part of the fragments, while the serosa part was placed in contact with the buffer. A schematic representation of the plates is presented in Figure 1. Subsequently, the plates were placed into an incubator shaker apparatus, SKI 4 Shaker Incubator (Argo Lab, Carpi, Italy), at 150 rpm and 25 °C.

At each predetermined time point (30, 60 and 120 min), the acceptor fluid (Krebs-Ringer bicarbonate buffer) was withdrawn from the wells and used for analysis. The samples withdrawn were evaporated (Büchi Rotavapor R-111, Westbury, NY, USA) at 70 °C, and acetonitrile was added to the solid in order to extract SS13. Several extractions were done to extract the maximum amount of SS13. The dispersion obtained was centrifuged for 10 min at 14,000 rpm; after filtration (0.22 µm cellulose regenerate filter), the supernatant was analyzed. The amount of permeated SS13 was determined using the HPLC method reported in Section 2.5. The results were expressed as percentage of SS13 permeated versus time (*n* = 3 ± standard deviation, SD).

To calculate the amount of unpermeated SS13, the residual formulations were recovered from the mucosa surface and analyzed. The residues were evaporated and dispersed in acetonitrile, and the suspensions, once centrifuged and filtered, were analyzed by HPLC. To determine the SS13 inside the mucosa, the intestinal fragments were washed and frozen to be used for further analysis. After thawing, the mucosa fragments were homogenized by an Ultra-Turrax IKA T25 (IKA, Staufen, Germany) with 5 mL of DCM. Successively, the suspension was subjected to centrifugation to precipitate the tissue residues, and the supernatant was evaporated. The solid obtained was mixed with acetonitrile, centrifuged, filtered and analyzed. The SS13 content recovered inside the mucosa and on the intestinal surface was expressed as a percentage with respect to the amount of SS13 in SLNs (*n* = 3 ± SD) [33].

Apparent permeability coefficients (*Papp*) were calculated with the following equation [34]:

(2) $$Papp=\frac{dc\times V\;}{\begin{array}{c} dt\times A\times Co \end{array}}$$

where *Papp* represents the apparent permeability expressed in cm/s, *dc*/*dt* is the molecule flux across the mucosa under steady-state conditions, *V* is the volume in the acceptor compartment, *A* is the mucosal surface area and *C*_0_ is the initial concentration of SS13 on the apical side.

### 2.13. In Vitro Cytotoxicity Evaluation

#### 2.13.1. Cell Culture

Caco-2 cells (European Collection of Cell Cultures (ECACC UK) (passage 20–40) were cultured in tissue culture flask T75 in Dulbecco’s modified Eagle’s medium (DMEM) with 10% FBS, 1% nonessential amino acid, 1% L-glutamine and 1% penicillin–streptomycin solution and incubated at 37 °C and 5% CO_2_.

#### 2.13.2. MTT Assay

The influence of different concentrations of SLN-W and SLN-G on Caco-2 cells was studied by MTT assay. A solution of SS13 in DMSO was used to evaluate the cytotoxicity of the compound alone. Cells were treated for 6 and 24 h with the two SLN formulations and the solution containing SS13 at 0.5, 1 and 3 µM. The SS13 concentrations were selected on the basis of a preliminary screening where several increasing concentrations of the molecule were evaluated. After 6 and 24 h contact, the MTT assay was carried out. For this, 1 mg/mL of MTT was added to each sample before incubation for 4 h at 37 °C. The soluble dye MTT is converted into the insoluble formazan crystals only by viable cells. After the end of incubation time, the solution of MTT was removed, the cells were washed with PBS and centrifuged and the pellet was dissolved in 2 mL of isopropanol. The absorbance was detected at 578 nm using a Bauty Diagnostic Microplate Reader. All tests, performed in 96-well plates (1 × 10^5^ cells/mL/well), were repeated in triplicate.

### 2.14. Statistical Analysis

To perform the statistical analysis, the GraphPad Prism 8.0 software (GraphPad Software, Inc., San Diego, CA, USA) was employed. The analysis of variance (one-way ANOVA) followed by Tukey’s multiple comparison test was used to analyze the data, and the significance level was set at *p* < 0.05.

## 3. Results and Discussion

As part of a long-lasting project, a new class of phenoxymethylquinoxalines capable of restoring or potentiating the antitumor activity of anticancer drugs in tumor cells by acting as EPIs was previously designed, synthesized and tested in *M. tuberculosis* strains. On this base, a new series of compounds were designed to be more selective against *M. tuberculosis* efflux pumps, to restore the activity of anti-TB drugs by reducing their cell extrusion. The most promising derivative was selected and labeled as SS13, and it was more widely tested on 10 *M. tuberculosis* strains.

### 3.1. Antimycobacterial Activity Assay

Compound SS13 was tested as an EPI in drug-association tests against a reference commercial *M. tuberculosis* strain (H37Rv) and nine clinically isolated strains. The therapeutic drugs used for the association tests with SS13 were four first-line drugs (streptomycin, isoniazid, rifampicin and ethambutol) and one fluoroquinolone (ciprofloxacin). Resazurin microtiter assay (REMA) was performed. Drug sensitivity testing was performed using the Bactec MGIT 960 automated mycobacterial detection system and three breakpoint concentrations of first-line antitubercular drugs: one strand was drug-sensitive (1670) and two strands were resistant to two drugs (368 and 1762), while four strands were MDR (512, 571, 600 and 1120) and three of them were resistant to all the tested antitubercular drugs (Table 1).

REMA test was used to test the 10 strands, as above. They were treated with ciprofloxacin in addition to the four first-line drugs. Ciprofloxacin showed activity against all strands except for 512 and 1670, against which it showed MIC values of 8 and 16 µg/mL, respectively. SS13 was tested alone on all the strains, and MICs were all > 512 µg/mL, proving a nonintrinsic antitubercular activity. As reported in Table 2, compound SS13 can increase the antitubercular activity of all the tested compounds on several strains. Streptomycin, rifampicin and ciprofloxacin are the most potentiated drugs when associated with our EPI compound, SS13. The MIC values of some drugs were reduced in resistant strains but in some cases also in sensitive strands. Strain 952 was sensitive when treated with INH and EMBm but the MIC values still decreased drastically when the drugs were associated with SS13. Strain 1670, as well as being sensitive to all the tested drugs, showed an increased sensitivity to the anti-TB drugs when combined with EPI SS13. MDR strains 512, 600 and 1120 showed how the drug resistance can be reverted when the strains are treated with a combination of anti-TB drug and EP inhibitor, such as our SS13.

### 3.2. Determination of SS13 Solubility and Partition Coefficient

Pharmacopeia describes the solubility of drugs as parts of solvent needed for a single part of solute (FU XII). It is also quantitatively expressed in terms of mass of solute that dissolves in a given volume of a solvent. Table 3 shows solubility results (in mg/mL and parts of solvent required for one part of solute) obtained for SS13. Although the pH influenced the solubility, the molecule was practically insoluble in all tested media. Indeed, as reported in the pharmacopeia, a drug substance is practically insoluble if its solubility is less than 0.1 mg/mL or if 1 part of solute requires more than 10,000 parts of solvent to dissolve it.

The analysis of Log P showed no SS13 in the aqueous phase; moreover, the compound amount found in the octanol phase was very low, and the Log P value was 0.817 ± 0.031 (mean of three determinations ± SD). This value indicates that the molecule does not have a high lipophilicity. Therefore, poor aqueous media solubility and low lipophilicity can limit the cell membrane permeation by passive diffusion [35,36].

As widely reported in the literature, the intestinal absorption of poorly water-soluble drugs is reduced, and their oral bioavailability is low [37,38,39]; therefore, their clinical application is greatly restricted. In this work, with the aim to enhance the oral bioavailability of SS13, some preliminary attempts have been made using some techniques that are usually employed to overcome disadvantages associated with poorly soluble drugs; in particular, the preparation of solid dispersion [40] and the complexation with cyclodextrins [41] were evaluated. Nevertheless, the results obtained were not good enough, and the solubility of SS13 was not increased (data not shown); this could be due to the negative chemical characteristics of the molecule itself. For example, in a recent paper, Ditzinger et al. suggested that most substances successfully formulated in solid dispersion had a Log P ranging from 2 to 4 [42]. Our study showed that the molecule was not partitioned between the lipid and aqueous phases; thus, SS13 probably does not have suitable characteristics for its formulation in solid dispersions. Therefore, these results support the necessity of formulating SS13 into a proper drug delivery system.

### 3.3. Preparation of SLNs

Two formulations with a different lipid composition were prepared. The dimensional analysis showed that, if compared with SLN-W (particle size and PDI of 450.6 nm and 0.141 respectively), the incorporation of G in the dispersion led to nanoparticles with higher values of mean diameter and lower suspension homogeneity (*p* < 0.05). After 30 s of sonication, the SLNs containing G showed a mean diameter of 620.8 nm and PDI of 1.261. The particle size is an important feature for the absorption rate of nanoparticles through the gastrointestinal tract: SLN dimensions in the range of 20–500 nm are suitable for absorption by intestinal cells [43]. Therefore, to decrease the diameter and the particle polydispersity, formulative studies were carried out and longer homogenization times were tested. When the sonication was increased from 30 to 60 s, a decrease in dimensions and PDI was observed (*p* < 0.05); besides, according to Das et al., prolonging the time of sonication to 90 s induced a further decrease in size and nanoparticles polydispersity (*p* < 0.05) (Table 4) [44]. Probably, this happened because longer sonication increased the energy, causing the disruption of coarse emulsion droplets to nanoemulsion drops and consequently decreasing the particle size [45]. On the basis of these results, for the preparation of W/G-based SLNs, 90 s was selected as sonication time. However, this reduction of mean diameter was possible only with the nanoparticles containing G. Indeed, concerning SLN-W, a further increase of sonication time led to phase separation; therefore, it was not possible to increase the sonication time over 30 s.

The nanoparticle preparation using G as lipid constituent was also investigated. However, it was not possible to obtain a stable emulsion: after the sonication, the separation of phases and the precipitation of SS13 were observed, indicating that G alone was not sufficient to obtain a stable SLN dispersion.

### 3.4. Determination of SS13 Loading

The percentage of SS13 loading of SLN-W and SLN-G was found to be 86.84 ± 2.49% and 100.00 ± 3.11%, respectively. The high loading capacity of SLNs could be attributed to the chemical composition of lipids. W and G consist of mono-, di- and triglyceride mixtures characterized by different chain lengths [46,47] that can offer imperfections where greater quantities of a drug can be accommodated. On the contrary, a lipid that mainly consists of similar molecules cannot entrap high amounts of a drug [48].

### 3.5. Analysis of Particle Size, Polydispersity and Zeta Potential of Loaded and Unloaded SLNs

Table 5 reports the dimensional properties and the zeta potential of unloaded and SS13-loaded SLNs. Nanoparticles containing the mixture of W/G, both loaded and unloaded, showed lower values of mean diameter when compared with SLNs containing only W (*p* < 0.05). Besides, the homogeneity of the suspension decreased when G was employed: SLN-Wb and SLN-W, although characterized by larger mean diameter values, showed PDI always lower than that of the corresponding sample containing G (0.213 ± 0.020 for SLN-Gb and 0.772 ± 0.051 for SLN-G) (*p* < 0.05). Unloaded SLNs showed a mean diameter ranging from 140 to 430 nm, whereas the particle size significantly increased for nanoparticles loaded with SS13 (*p* < 0.05). The results obtained are in good agreement with those described by Sandri et al. which reported how a modification of surfactant and lipid rearrangement could be determined by drug entrapment in the lipid matrix, and this may cause an increase in mean diameter [49]. No significant differences were found between PDI of SLN-W and SLN-Wb (*p* > 0.05); in contrast, SLN-G displayed a higher nanoparticle polydispersity than SLN-Gb (*p* < 0.05). Although both formulations had a particle size of less than 500 nm (and therefore suitable for intestinal absorption), SLN-G, which was characterized by a mean diameter of about 250 nm, showed the best dimensional properties for the absorption by intestinal cells. However, the PDI was around 0.7, indicating that the particles were in a state of polydispersity distribution [50]. This is probably because, in aqueous solvents, G may undergo solubilization or dispersion with consequent formation of vesicles, micelles or microscopic globules [51] that could lead to the presence of multiple nanoparticle populations. However, it was observed that more than 71% of the nanoparticle population showed a particle size of 146.0 nm, suitable for intestinal uptake.

Both loaded and unloaded SLNs exhibited negative values of zeta potential, which ranged from −10.52 to −17.13 mV. The presence of G in formulation composition increased the negative charge of nanoparticle surfaces, but only in unloaded SLNs (SLN-Gb) (*p* < 0.05). Moreover, no statistical differences were found after the loading process (*p* > 0.05). Based on these results, we may suppose that SS13 is entrapped in SLNs. Both loaded SLNs showed a negative surface charge, and SS13 also had a negative zeta potential (−9.24 ± 0.84). If this molecule was adsorbed onto the SLN surface, an increase in negative charge would be expected. As we could not detect any significant difference between loaded and unloaded nanoparticles, an encapsulation of SS13 could be hypothesized.

### 3.6. Morphological Analysis

#### 3.6.1. Transmission Electron Microscopy

**Figure 2:**
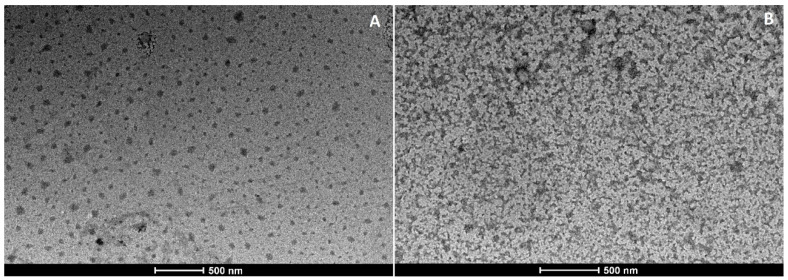
TEM images of SLN-W (**A**) and SLN-Wb (**B**).

**Figure 3:**
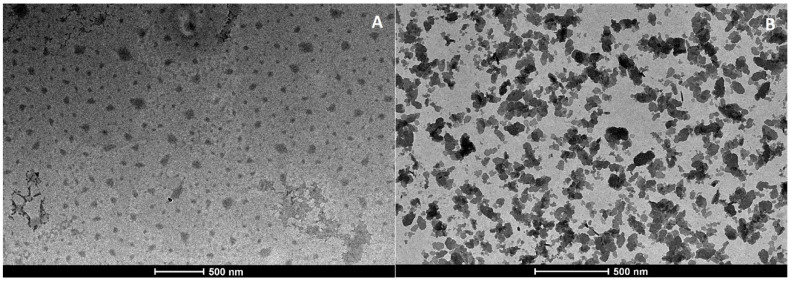
TEM images of SLN-G (**A**) and SLN-Gb (**B**).

Figure 2 and Figure 3 show TEM images of loaded and unloaded SLNs. Particle aggregation was observed in both unloaded SLNs, even if a different morphology was evident. SLN-Wb appeared to be a single cluster of nanoparticles (Figure 2B), while the aggregates were separated in SLN-Gb (Figure 3B). The aggregation was probably due to the drying procedure [33]. On the contrary, loaded nanoparticles looked isolated and exhibited a spherical shape (Figure 2A and Figure 3A), suggesting that the aggregation process could be reduced by the presence of SS13. Nevertheless, in SLN-G, some larger particles could be observed; this corroborates the PDI results, showing different nanoparticle populations.

#### 3.6.2. Atomic Force Microscopy

**Figure 4:**
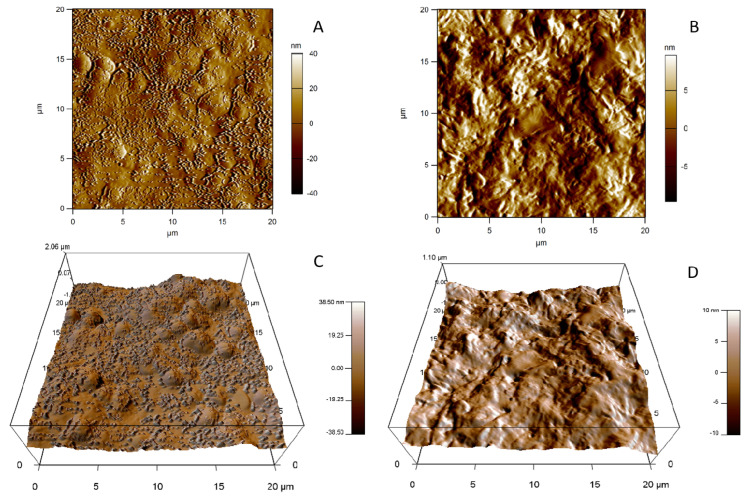
Topographic images of SLN-W (**A**) and SLN-Wb (**B**). Three-dimensional views of 20 × 20 µm scan of SLN-W (**C**) and SLN-Wb (**D**).

**Figure 5:**
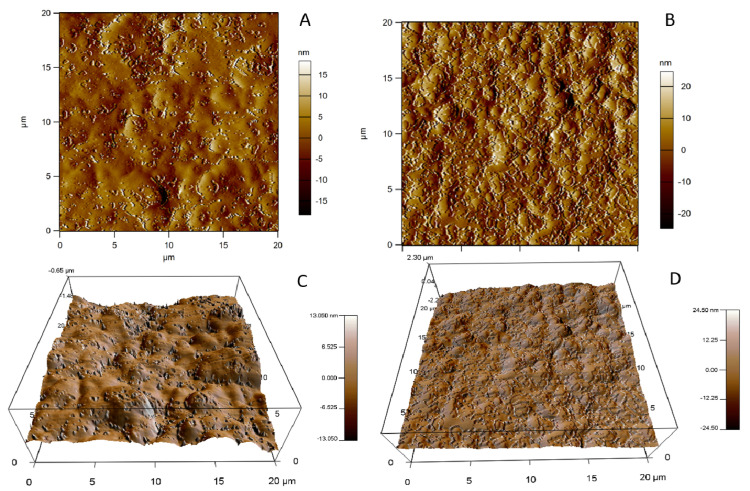
Topographic images of SLN-G (**A**) and SLN-Gb (**B**). Three-dimensional views of 20 × 20 µm scan of SLN-G (**C**) and SLN-Gb (**D**).

AFM micrographs of unloaded and SS13-loaded SLNs are shown in the figures below. The results are in agreement with the TEM observations: unloaded SLNs were composed of clusters of nanoparticles, and morphological differences were observed between SLN-Wb (Figure 4B,D) and SLN-Gb (Figure 5B,D). AFM images of SLN-W (Figure 4A,C) and SLN-G (Figure 5A,C) showed nanoparticles with similar morphology, and it was possible to observe roundish particles.

### 3.7. Fourier Transform Infrared Spectrometry Analysis

**Figure 6:**
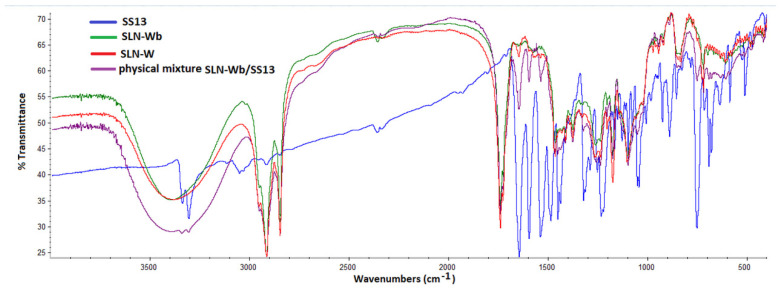
FTIR spectra of SS13, SLN-Wb, SLN-W and physical mixture of SLN-Wb and SS13.

**Figure 7:**
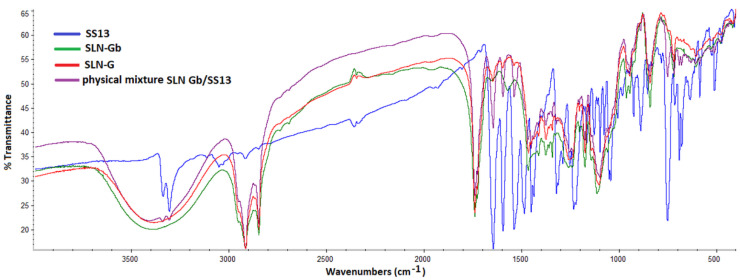
FTIR spectra of SS13, SLN-Gb, SLN-G and physical mixture of SLNGb and SS13.

Figure 6 and Figure 7 show the FTIR spectra of loaded and unloaded SLNs and SS13 alone. Band positions for various groups were assigned based on those previously reported in the literature. Both spectra revealed characteristic peaks at 2917 and 2850 cm^−1^ (C-H stretching), 1741 cm^−1^ (C=O stretching) and 1115 cm^−1^ (C-O stretching) owing to the lipid content in the formulation. The absorption band in the range of 3300–3450 cm^−1^ was from O-H stretching of PVA [52,53,54]. The peaks in the 1647–1597 cm^−1^ and 1540–1487 cm^−1^ regions are due to C-C stretching vibrations in the aromatic ring [55,56] and are typical of SS13. The absence in SLN-W and SLN-G FTIR spectra of peaks of the aromatic ring could be a further confirmation of SS13 entrapment in the SLN; on the contrary, these peaks were observed for the physical mixtures.

### 3.8. X-ray Analysis

**Figure 8:**
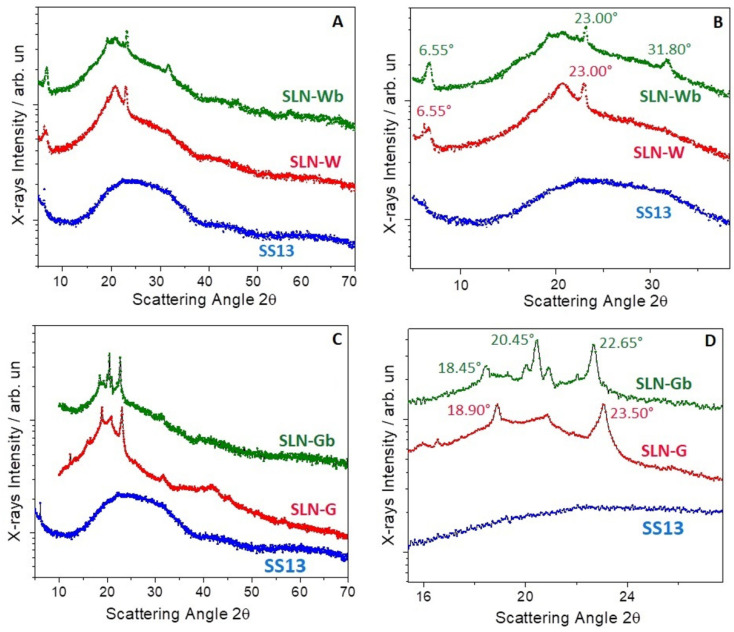
XRD pattern of SLNs prepared with Witepsol E85 (W) (**A**,**B**) and with the mixture of W/G (**C**,**D**). Magnification of XRD pattern of SLNs with W (**B**) and with W/G (**D**).

The XRD pattern of SS13 indicated that the compound had an amorphous structure, while crystal diffraction peaks appeared in SLN formulations, both loaded and unloaded, probably due to a lipid crystallization during the process of solvent evaporation. The XRD graph in Figure 8C,D shows that the amorphous peak of SS13 disappeared completely in SLN-G, indicating that the molecule is probably dispersed in the nanoparticles. Indeed, compared to SLN-Gb, SLN-G peaks are different and shifted to higher angles.

On the contrary, concerning SLN-W, the peaks of SLN-Wb, as well as the amorphous peak of SS13, are still evident (Figure 8A,B).

### 3.9. Physical Stability Studies

**Figure 9:**
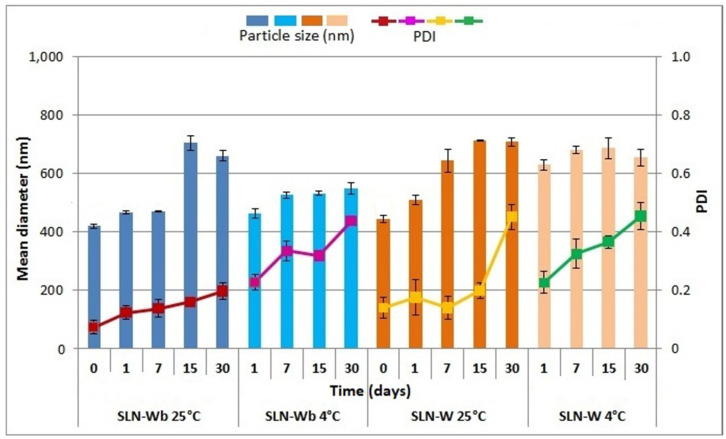
Influence of storage (at 4 and 25 °C) on the particle size and PDI of SLN-Wb and SLN-W. Particle size: SLN-Wb at 25 °C, 0 days vs. 15 and 30 days (*p* < 0.05); SLN-Wb at 4 °C, 0 days vs. 7, 15 and 30 days (*p* < 0.05); SLN-W at 25 °C, 0 days vs. 7, 15 and 30 days (*p* < 0.05); SLN-W at 4 °C, 0 days vs. 1, 7, 15 and 30 days (*p* < 0.05). PDI: SLN-Wb at 25 °C, 0 days vs. 7, 15 and 30 days (*p* < 0.05); SLN-Wb at 4 °C, 0 days vs. 1, 7, 15 and 30 days (*p* < 0.05); SLN-W at 25 °C, 0 days vs. 30 days (*p* < 0.05); SLN-W at 4 °C, 0 days vs. 7, 15 and 30 days (*p* < 0.05).

Figure 9 shows the influence of time and storing conditions on the stability trend of SLNs prepared with W. SLN-Wb was stable for the first 2 weeks when stored at 25 °C (*p* > 0.05), and the mean diameter increased from 425.1 nm on day 0 to 705.0 nm on day 15 (*p* < 0.05). However, at 4 °C, nanoparticle aggregation occurred at 7 days (*p* < 0.05). The storing conditions also influenced the particle size distribution, and a lower dispersion homogeneity was observed at 4 °C. Indeed, in terms of PDI, at room temperature, no significant variations were observed from day 0 to day 7 (*p* > 0.05), while an increase after day 1 was noted for formulations at 4 °C (*p* < 0.05). Drug loading made the SLNs more stable in terms of particle distribution, but nanoparticle aggregation was noted to occur more quickly. When stored at room temperature, the PDI did not change before day 30 (*p* < 0.05), although the particle size increased to over 600 nm after 7 days (*p* < 0.05). At 4 °C, the particle diameter increased over time, reaching 630.9 nm after 1 day (*p* < 0.05), while the PDI changed from 0.14 to 0.33 after 1 week (*p* < 0.05).

**Figure 10:**
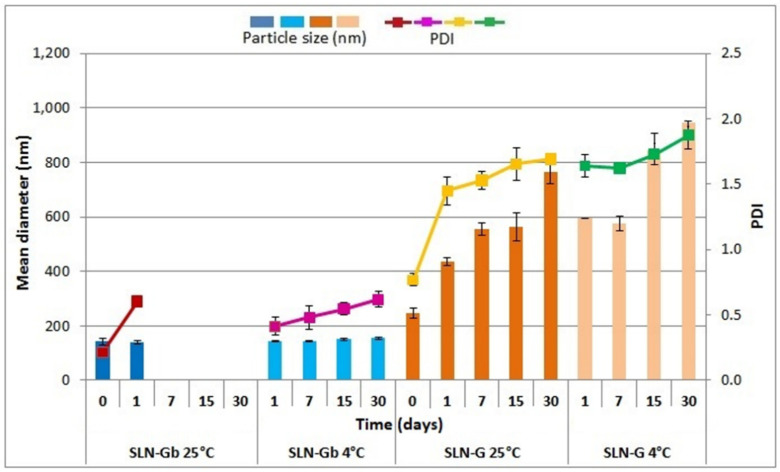
Influence of storage (at 4 and 25 °C) on the particle size and PDI of SLN-Gb and SLN-G. Particle size: SLN-G at 25 °C, 0 days vs. 1, 7, 15 and 30 days (*p* < 0.05); SLN-G at 4 °C, 0 days vs. 1, 7, 15 and 30 days (*p* < 0.05). PDI: SLN-Gb at 25 °C, 0 days vs. 1 day (*p* < 0.05); SLN-Gb at 4 °C, 0 days vs. 1, 7, 15 and 30 days (*p* < 0.05); SLN-G at 25 °C, 0 days vs. 1, 7, 15 and 30 days (*p* < 0.05); SLN-G at 4 °C, 0 days vs. 1, 7, 15 and 30 days (*p* < 0.05).

Studies on SLNs based on the lipid mixture of W/G demonstrated that nanoparticle stability was influenced both by the formulation composition and the temperature (Figure 10). The effect of storage condition on particle stability was more evident in unloaded SLNs. If stored at 25 °C, the mean diameter of SLN-Gb did not increase from day 0 to day 1 (*p* > 0.05), but it was not possible to perform subsequent dimensional analysis because the emulsion broke and separation into the aqueous and oil phases took place. On the contrary, at 4 °C, particle size was not modified over time (*p* > 0.05), though the PDI increased from 0.42 to 0.62 after 1 day (*p* < 0.05). Regarding SLN-G, both at 4 and 25 °C, an increase in mean diameter and PDI was observed on day 1 (*p* < 0.05).

Nanoparticles containing W alone, both loaded and unloaded, were stable in terms of particle size and dispersion homogeneity for 7 days if stored at 25 °C, while less stability was found at 4 °C. In contrast, the particles with the W/G mixture showed poor stability; in particular, the loaded SLNs underwent a quite rapid increase of mean diameter and PDI values. The presence of aggregates that develop in a short time in systems prepared with G has been observed [46]. This effect could be due to a partial lipid matrix rearrangement. G is a mixture of mono-, di- and triglycerides [48]. These glycerides are characterized by three polymorphs; therefore, it is probable that, during storage, the conversion from one polymorph into another occurred. This could reduce the space in the lipid matrix, causing the presence of a small amount of SS13 on the surface or outside of the SLNs. This could lead to intra- or intermolecular interaction, causing a reduction in physical stability [57,58].

As previously reported, particle size is an important parameter for nanoparticle uptake by intestinal cells. Therefore, to determine whether the SLNs can reach the intestine intact, their stability was also evaluated in buffer pH 1.2 for 2 h. The test showed that no dimensional variation occurred, indicating that the SLNs remain stable in simulated gastric acidic conditions. Moreover, physical changes such as agglomeration, phase separation and precipitation were not observed.

### 3.10. In Vitro SS13 Release Study

The solubility of SS13 in different media (Tween/PBS and FBS/PBS) was determined in order to select the suitable release media [29]. SS13 showed the solubility of 0.00219 mg/mL in Tween/PBS and 0.00811 mg/mL in FBS/PBS; thus, FBS/PBS was chosen to perform in vitro release study.

**Figure 11:**
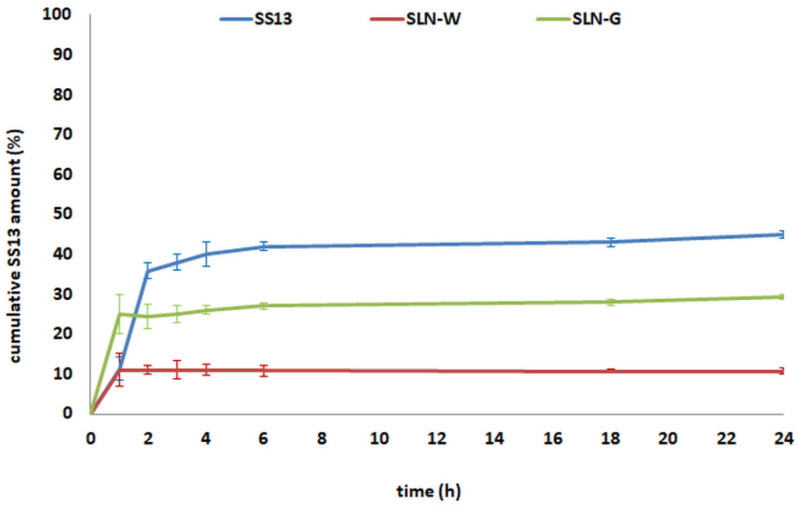
In vitro release kinetics of SS13 from SLN-W and SLN-G compared to the dissolution of free molecule. Data are reported as mean ± SD (*n* = 3).

Results from in vitro dissolution study (Figure 11) showed that 35% of SS13 dissolved in the medium within 2 h; then, the dissolution rate decreased and 45% was found in the release medium after 24 h. Concerning SLN formulations, an initial burst was observed, and 11% and 24% of SS13 was released from SLN-W and SLN-G, respectively. Afterward, the cumulative SS13 amount did not change for up to 24 h. This behavior may be attributed to the solubilizing effect of serum proteins [29] of the small amount of SS13 present in the external portion of the SLN lipid matrix.

### 3.11. Ex Vivo Permeation Studies on Intestinal Mucosa

**Figure 12:**
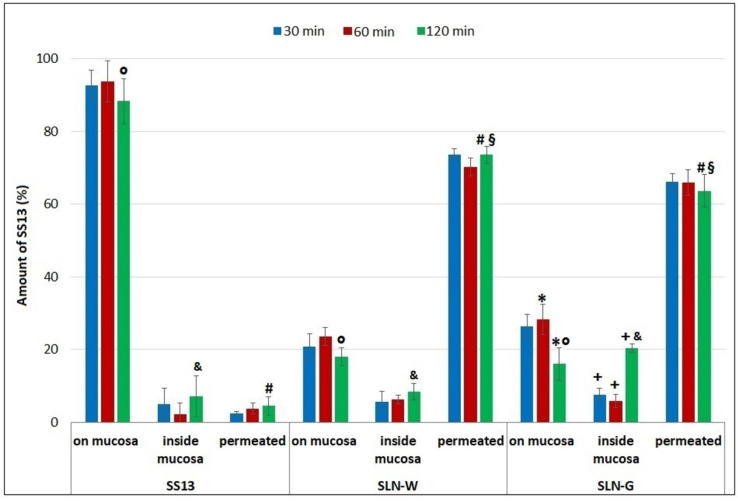
Ex vivo distribution of SS13 during the permeation test (30, 60 and 120 min) through the intestinal mucosa (*n* = 3). * *p* < 0.05, SLN-G on mucosa 120 min vs. SLN-G on mucosa 60 min; ^+^ *p* < 0.05, SLN-G inside mucosa 120 min vs. SLN-G inside mucosa 30 and 60 min; ^°^ *p* < 0.05, SS13 on mucosa 120 min vs. SLN-W on mucosa 120 and SLN-G on mucosa 120 min; ^&^ *p* < 0.05, SLN-G inside mucosa 120 min vs. SLN-W inside mucosa 120 min and SS13 inside mucosa 120 min; ^#^ *p* < 0.05, SS13 permeated 120 min vs. SLN-W permeated 120 min and SLN-G permeated 120 min; ^§^ *p* < 0.05, SLN-W permeated 120 min vs. SLN-G permeated 120 min.

Figure 12 reports the amount of SS13 permeated and that determined on and inside the intestinal mucosa at different time points (30–120 min) during the ex vivo permeation test. Due to its water insolubility and low Log P, SS13 was not able to permeate the intestinal tissue, and at the end of the test, most of this molecule was recovered on the mucosa surface; indeed, it is known that molecules with low Log *p* values show poor ability to pass through the cell membrane [35,36]. On the contrary, both the SLN formulations favored the SS13 transport through the intestinal mucosa and showed a rapid permeation profile: after 30 min, about 74% of SS13 was found in the acceptor medium of SLN-W, and 66% of SS13 was found in that of SLN-G. This is further confirmed by the greatest values of the apparent permeability coefficient of the SLN formulations (Table 6). The amount of SS13 permeated did not change during the time, both for SLN-W and SLN-G (*p* > 0.05); therefore, if compared with SS13 in dispersion, the SLNs facilitated more rapid and efficient permeation across the intestinal tissue. The increment in the permeation of SS13 obtained with the SLNs may be due to the reduction in particle dimensions and improvement of surface area that led to a greater rate of dissolution and diffusion [59].

In addition, these results demonstrated that SLN-G supported the uptake of SS13 into the intestinal mucosa; indeed, after 120 min, 25% of SS13 was found inside the tissue. On the contrary, low percentages of this compound from SS13 dispersion and SLN-W (11.26% and 12.66% respectively) were found in the mucosa after the permeation test. This may be due to the solubility/dissolution rate enhancing effect of G itself [60,61] that may improve the intestinal absorption. Furthermore, as reported by Langasco et al., the surface negative electrical charge of SLNs could be a positive characteristic for the internalization of the particle by nonphagocytic cells (such as enterocytes) [62]. The lower cellular uptake observed for SLN-W (despite its negative zeta potential), could suggest a different intestinal transport mechanism for the two formulations: transcellular for SLN-G and paracellular for SLN-W. Further studies, based on the use of inhibitors of the steps involved in internalization routes, are needed in order to define the specific transport pathway [63,64].

### 3.12. In Vitro Cytotoxicity Evaluation

#### MTT Assay

**Figure 13:**
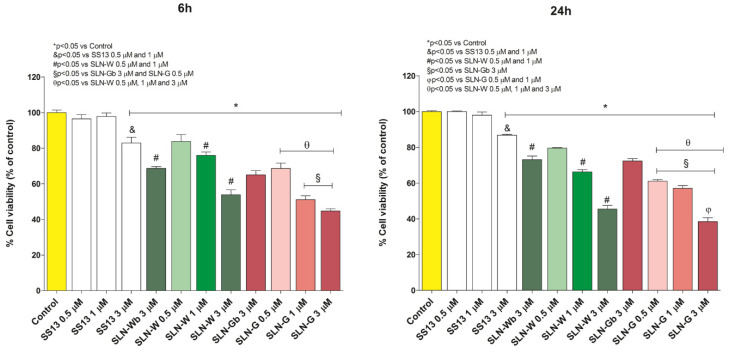
Effect of increasing concentrations (0.5, 1 and 3 µM) of SS13, SLN-Wb, SLN-W, SLN-Gb and SLN-G on CACO-2 cells at 6 and 24 h of exposure. Data are reported as mean ± SD (n = 3). * *p* < 0.05 vs. Control; ^&^ *p* < 0.05 vs. SS13 0.5 and 1 µM; ^#^ *p* < 0.05 vs. SLN-W 0.5 and µM; ^§^ *p* < 0.05 vs. SLN-Gb 3 µM and SLN-G 0.5 µM; ^φ^ *p* < 0.05 vs. SLN-G 0.5 and 1 µM; ^θ^ *p* < 0.05 vs. SLN-W 0.5, 1 and 3 µM.

Figure 13 represents the effects of different concentrations of SS13, SLN-Wb, SLN-W, SLN-Gb and SLN-G on Caco-2 cells at 6 and 24 h of exposure.

The analysis demonstrates that all nanoparticles tested show good biocompatibility at all concentrations of treatment with the exception of SLN-Gb and SNL-G, which induce an increase of cell death at 3 µM concentration. All nanoparticles analyzed show a moderate decrease of the cell viability until 1 µM concentration, suggesting an increase of the cell death proportional to the increase of the dose administrated.

## 4. Conclusions

SS13 improved the activity of different antitubercular drugs on several *M. tuberculosis* strains and could be a good candidate in the fight against antimicrobial resistance; however, SS13 showed low solubility in simulated gastrointestinal media and a low Log P value, both of which can affect oral absorption. The use of nanotechnology was found to be an efficient strategy to improve intestinal drug permeation: SLNs, based on different lipid compositions, were successfully prepared using a solvent evaporation method. Formulation composition influenced the particle size as well as the physical stability of SLNs, which showed a good loading ability. The nanoparticles affected Caco-2 viability in a dose-dependent manner and were able to increase the ex vivo permeation of SS13 through the intestinal mucosa with regard to their composition. In particular, SLN-G showed the highest loading capacity, a suitable particle size and the capability to increase the intestinal permeation as well as the mucosal uptake of SS13. These promising results suggest that SLN-G offers an encouraging approach for increasing the oral permeation of SS13 in multidrug-resistant tuberculosis management.

## Figures and Tables

**Table 1:** Drug sensitivity test results for *M. tuberculosis* strains used for the following resazurin microtiter assay (REMA): sensitive (S) and resistant (R). In the last column, the level of resistance is assigned as drug-resistant (DR) or multidrug-resistant (MDR). SM: streptomycin; INH: isoniazid; RIF: rifampicin; EMB: ethambutol.

Strain Label	SM	INH	RIF	EMB	Res
H37Rv	S	S	S	S	
952	S	S	R	S	DR
1097	R	R	S	R	DR
1670	S	S	S	S	
512	S	R	R	R	MDR
600	R	R	R	R	MDR
368	S	R	S	R	DR
1762	S	S	R	R	DR
571	R	R	R	R	MDR
1120	R	R	R	R	MDR

**Table 2:** REMA test results for 10 *M. tuberculosis* strains treated with antitubercular drugs alone and in association with SS13 (SS13). MICs reported are given in µg/mL. In round brackets, the SS13 concentration needed to get the correspondent MIC of each drug in the same cell (µg/mL) is shown. Symbols represent the drug activity, which was increased (MIC reduced) x-fold by SS13 effect: **A**, x = 0.8; **B**, x = 2; **C**, x = 4; **D**, x = 8; **E**, x = 16; **F**, x = 64.

Strand Label	SM	SM+(SS13)		INH	INH+(SS13)		RIF	RIF+(SS13)		EMB	EMB+(SS13)		CIPR	CIPR+(SS13)	
H37Rv	0.125	0.125		0.125	0.125		0.25	0.06(128)	**C**	1	1		0.5	0.5	
952	0.062	0.062		0.062	0.0078(32)	**D**	0.5	0.062(32)	**D**	1	0.25(64)	**C**	2	0.125(16)	**E**
1097	4	0.06(8)	**F**	2	2		0.25	0.062(128)	**C**	32	32		0.5	0.125(64)	**C**
1670	0.125	0.06(128)	**B**	0.125	0.125		0.125	0.062(128)	**B**	1	1		16	<0.25(4)	**F**
512	0.5	0.031(128)	**E**	1	1		>2	>2		4	1(32)	**C**	8	4(64)	**B**
600	>4	1(25)	**C**	>1	0.25(25)	**C**	>2	0.5(25)	**C**	>16	16(100)		4	1(50)	**C**
368	0.5	0.06(6.25)	**D**	<1	0.125(3.125)	**D**	0.25	0.125(25)	**B**	>16	2(3.125)	**D**	0.5	0.25(25)	**B**
1762	0.125	0.015(6)	**D**	0.062	0.078(6)	**A**	>2	>2		4	0.25(3)	**E**	1	0.5(25)	**C**
571	0.25	0.25		>1	>1		>2	>2		>16	>16		1	1	
1120	4	0.25(12.5)	**E**	>16	>16		8	4(200)	**B**	>64	>64		0.5	0.25(200)	**B**

**Table 3:** Solubility of SS13 obtained in different media.

Medium	Solubility (mg/mL)	Parts of Solvent Required for One Part of Solute	Solubility Definition (FU XII)
Bidistilled water	0.00113	888,489	Practically insoluble
Buffer pH 1.2	0.00459	217,946	Practically insoluble
Buffer pH 6.8	0.00128	1,119,224	Practically insoluble
Buffer pH 7.4	0.00145	633,583	Practically insoluble

**Table 4:** Influence of sonication time on the particle size and polydispersity index (PDI) of the formulation containing Gelucire 44/14 (G).

Time (s)	Mean Diameter (nm)	PDI
30	620.8 ± 21.1	1.261 ± 0.062
60	494.6 ± 16.2 ^#^	0.967 ± 0.084 ^§^
90	247.1 ± 19.7 ^#^*	0.772 ± 0.051 ^§+^

^#^*p* < 0.05 vs. 30 s; * *p* < 0.05 vs. 60 s; ^§^ *p* < 0.05 vs. 30 s; ^+^ *p* < 0.05 vs. 60 s.

**Table 5:** Average particle size, PDI and zeta potential of prepared solid lipid nanoparticles (SLNs). Results are expressed as mean ± SD.

Formulation	Mean Diameter (nm ± SD)	PDI (±SD)	Zeta Potential (mV ± SD)
SLN-Wb	425.1 ± 10.3	0.071 ± 0.021	−10.72 ± 1.01
SLN-Gb	141.8 ± 12.0 *	0.213 ± 0.020 ^a^	−17.13 ± 2.51 °
SLN-W	450.6 ± 14.9 ^+^	0.141 ± 0.030	−10.52 ± 2.33
SLN-G	247.1 ± 19.8 ^§#^	0.772 ± 0.051 ^bc^	−13.82 ± 2.44

* *p* < 0.05 vs. SLN-Wb; ^§^ *p* < 0.05 vs. SLN-W; ^+^ *p* < 0.05 vs. SLN-Wb; ^#^ *p* < 0.05 vs. SLN-Gb; ^a^ *p* < 0.05 vs. SLN-Wb; ^b^ *p* < 0.05 vs. SLN-W; ^c^ *p* < 0.05 vs. SLN-Gb; ° *p* < 0.05 vs. SLN-Wb.

**Table 6:** Apparent permeability coefficient of SS13, SLN-W and SLN-G.

Sample	Papp (cm/s)
SS13	4.57 × 10^−8^
SLN-W	8.61 × 10^−5^
SLN-G	8.23 × 10^−5^
